# LINC00472 suppressed by ZEB1 regulates the miR‐23a‐3p/FOXO3/BID axis to inhibit the progression of pancreatic cancer

**DOI:** 10.1111/jcmm.16784

**Published:** 2021-08-07

**Authors:** Cong Bi, Gang Wang

**Affiliations:** ^1^ Department of Radiology The First Affiliated Hospital of China Medical University Shenyang China; ^2^ Interventional Department The Fourth Affiliated Hospital of China Medical University Shenyang China

**Keywords:** BH3‐interacting domain death agonist, forkhead box O3, long intergenic non‐protein coding RNA 00472, microRNA‐23a‐3p, pancreatic cancer, zinc finger E‐box binding homeobox 1

## Abstract

The tumour‐suppressive role of LINC00472 has been extensively reported in various human cancers such as lung, colon and ovarian cancers, yet its function in pancreatic cancer remains unidentified. Here, the current research aimed to explore the role and regulatory axis mediated by LINC00472 in the progression of pancreatic cancer. RT‐qPCR was adopted to determine LINC00472 expression in the harvested pancreatic cancer tissues and adjacent normal tissues. Loss‐of‐function and gain‐of‐function experiments were performed to examine the effects of LINC00472 on proliferation and apoptosis in vitro and tumorigenesis in vivo. Immunoblotting was performed to detect the expression of several proliferation and apoptosis‐related proteins. Bioinformatic analysis, dual‐luciferase reporter assay and RNA pull‐down were conducted to profile the relationships between LINC00472 and miR‐23a‐3p, between miR‐23a‐3p and FOXO3 and between FOXO3 and BID. The LINC00472 expression was down‐regulated by ZEB1 in the pancreatic cancer cells and tissues. LINC00472 could competitively bind to miR‐23a‐3p to enhance the expression of FOXO3, which consequently could promote the BID expression, thereby suppressing proliferation and promoting the apoptosis of pancreatic cancer cells. Meanwhile, the inhibitory role of LINC00472 in tumorigenesis was validated in vivo, and the LINC00472‐mediated miR‐23a‐3p/FOXO3/BID axis was also demonstrated in the nude mouse tumour formation model. The study substantiated the antitumour activity of LINC00472 in pancreatic cancer and proposed a regulatory axis in which LINC00472 competitively binds to miR‐23a‐3p to enhance the FOXO3 expression and promote BID expression. Consequently, these findings provide theoretical basis for developing potential targets for the treatment of pancreatic cancer.

## 1 INTRODUCTION

Pancreatic cancer is regarded as the 17th leading cause of cancer‐related death worldwide as indicated by statistics. Currently, the prognostic outcome for pancreatic cancer patients is certainly poor with an insignificant 5‐year survival rate of around 10%.^1^, ^2^, ^3^ The high mortality of pancreatic cancer is associated with late diagnosis, as in the early stages, pancreatic cancer tends to be asymptomatic and only becomes symptomatic upon progression into advanced or metastatic stages.^4^ As for the risk factors for pancreatic cancer, intrinsic parameters such as elder age, family history and genetic susceptibility are the chief non‐modifiable risk factors, while parameters such as smoking, alcohol consumption and obesity have been extensively investigated and verified as the most significant modifiable risk factors.^5^ Over decades, a combination approach with surgical resection and chemotherapy or radiation therapy has persisted as the gold standard treatment protocol for pancreatic cancer patients as only a limited number are localized, surgically resectable tumours.^6^, ^7^ For future advancements in this field, investigating the development pathways of pancreatic cancer is of great significance for identifying novel diagnostic biomarkers and therapeutic targets to improve the prognosis of patients with pancreatic cancer.

Long intergenic non‐protein coding RNAs (lincRNAs) are a set of autonomously transcribed non‐coding RNAs with transcript lengths exceeding 200 nucleotides, and this group constitutes over 50% of all long non‐coding RNA (lncRNA) transcripts in humans.^8^ LincRNA 00472 (LINC00472) has demonstrated functionality as a competing endogenous RNA (ceRNA) to bind to several microRNAs (miRNAs) to suppress the progression of multiple types of cancers, including lung cancer^9^ and colorectal cancer.^10^ Additionally, miRNA has demonstrated to have vital significance in the development of human cancers by post‐transcriptional regulation of tumour suppressors or promoters,^11^ and miR‐23a‐3p could exercise a tumour‐promoting effect on pancreatic cancer by targeting the transforming growth factor‐β receptor type II (TGFBR2).^12^ Moreover, forkhead box O3 (FOXO3) was a tumour suppressor targeted by miRNA in human pancreatic cancer progression,^13^ while FOXO3a has been previously demonstrated to regulate BH3‐interacting domain death agonist (BID), which is a pro‐apoptotic protein belonging to the B‐cell lymphoma‐2 (Bcl‐2) protein family.^14^ An existing study elicited BID as an essential regulator of cell apoptosis in pancreatic cancer.^15^ As we predicted that FOXO3 was a target of miR‐23a‐3p and LINC00472 could bind to miR‐23a‐3p based on the bioinformatic analysis, we speculated the functionality of LINC00472 as a ceRNA of miR‐23a‐3p to regulate FOXO3 and subsequently influence BID‐dependent cancer cell apoptosis. In addition, Zinc finger E‐box binding homeobox 1 (ZEB1) has been demonstrated to interact with lncRNAs to regulate their transcription and affect pancreatic cancer migration, invasion and epithelial‐mesenchymal transition,^16^ suggesting that ZEB1 may be involved in the cell apoptotic pathway initiated by LINC00472. In this study, we constructed both cellular and animal models to systematically investigate the signalling axis ZEB1/LINC00472/miR‐23a‐3p/FOXO3 in pancreatic cancer.

## 2 MATERIALS AND METHODS

### 2.1 Ethics statement

All experimental procedures were conducted with approval of the Ethics Committee of The Fourth Affiliated Hospital of China Medical University and in conformity with the *Declaration of Helsinki*. All patients and/or legal guardians signed informed consent documentation prior to enrolment. All animal experiments were performed with approval of the Animal Experiment Ethics Committee of The Fourth Affiliated Hospital of China Medical University and conducted in strict accordance with the Guide for the Care and Use of Laboratory Animals issued by the US National Institutes of Health. Adequate measures were taken to minimize the number of animals used in the experiments and their suffering.

### 2.2 Microarray‐based gene expression profiling

Pancreatic cancer‐related gene expression data sets GSE46234 and GSE24279 were retrieved from the Gene Expression Omnibus (GEO) database (https://www.ncbi.nlm.nih.gov/geo/), with the probe file downloaded using the Affymetrix Human Genome U133 Plus 2.0 Array and febit human miRBase v11. GSE46234 data set comprised of 4 normal samples and 4 tumour samples, while the GSE24279 data set comprised of 22 normal samples and 136 tumour samples. With the normal sample as control, the Affy installation package of R software was used for background correction and normalization on each data set.^17^ Non‐specific filtering of the expression profile data was conducted by the linear models, while the screening of the differentially expressed lncRNAs and miRNA was conducted by a combination of the empirical Bayes methods in the Limma installation package and the traditional *t* test.^18^ The starBase database (http://starbase.sysu.edu.cn/) was adopted to predict the miRNA bound by lncRNA and the target gene of miRNA.^19^

### 2.3 Collection of clinical samples

Pancreatic cancer tissues and adjacent normal tissues were obtained from 70 patients (42 males and 28 females) with a mean age of 59 years, who underwent surgery at The Fourth Affiliated Hospital of China Medical University between a period from March 2012 to March 2014. The collected tissue samples were preserved at −80°C for further experimental assays. All cases were histologically verified as pancreatic cancer by three independent histopathologists. Tumours were graded and classified according to the American Joint Committee on Cancer Staging Manual (Table S1). An investigation was conducted for the expression of LINC00472 in the pancreatic tissues and adjacent normal tissues by reverse transcription‐quantitative polymerase chain reaction (RT‐qPCR). Subsequently, the median LINC00472 expression in the pancreatic cancer tissues was used as cut‐off to distinguish between the cancer tissues exhibiting high and low expression of LINC00472.

### 2.4 Follow‐up

The participants were followed up by telephone or on appointment basis. The follow‐up deadline was February 2019. The overall survival (OS) of the patients was defined as the time starting from randomization until the death of patient caused by any reason. The 5‐year OS of patients was calculated. As of the follow‐up deadline, a total of five patients were during the follow‐up period among the initial 70 patients, resulting in a follow‐up rate of 89.55%.

### 2.5 Cell culture and treatment

The normal immortalized human pancreatic epithelial cell line hTERT‐HPNE (ATCC^®^ CRL‐4023) and four human pancreatic cancer cell lines SW1990 (ATCC^®^ CRL‐2172), BXPC3 (ATCC^®^ CRL‐1687), Capan‐2 (ATCC^®^ HTB‐80) and PANC‐1 (ATCC^®^ CRL‐1469) were provided by the American Type Culture Collection (ATCC; Manassas, VA, USA). All cells including the HEK‐293T (Cell Resource Center, Shanghai Institutes for Biological Sciences, Chinese Academy of Sciences) were cultured in Dulbecco's modified Eagle's medium (DMEM) (Sigma‐Aldrich, St. Louis, MO, USA) supplemented with 10% foetal bovine serum (FBS; HyClone, Logan, UT, USA) and reserved in a humidified incubator with 5% CO_2_ at 37°C.

Lentiviral vectors overexpressing ZEB1 (oe‐ZEB1), LINC00472 (oe‐LINC00472), FOXO3 (oe‐FOXO3), BID (oe‐BID) and their LINC00472 controls (oe‐NC) were acquired from GenePharma (Shanghai, China). The miR‐23a‐3p mimic and its NC, and short hairpin RNA (shRNA) against LINC00472 (sh‐LINC00472) were provided by RiboBio (Guangzhou, China). The shRNA sequences are shown in Table 1. For cell transfection, the cells were grown in 6‐well plates until they attained 60% confluence, after which the cells were transfected with miRNA mimic or shRNA, or infected with the lentiviral vectors using the Lipofectamine 3000 kit (Invitrogen, Carlsbad, CA, USA).

**TABLE 1:** shRNA sequences

shRNAs	Sequence
sh‐LINC00472#1	5′‐GGAATCTTTCTCAGTCCTTAG‐3′
sh‐LINC00472#2	5′‐GCAACAGAAGTATGTGCAAGA‐3′
sh‐LINC00472#3	5′‐GCTAAGGGTAGACTCATATTT‐3′
sh‐NC	5′‐TTCTCCGAACGTGTCACGTTT‐3′

Abbreviations: LINC00472, long intergenic non‐protein coding RNA 472; NC, negative control; sh/shRNA, short hairpin RNA.

### 2.6 RT‐qPCR

The total RNA content was extracted using a RNeasy Mini Kit (74104, Qiagen, Valencia, CA, USA) and reverse‐transcribed into complementary DNA (cDNA) using the reverse transcription kit (RR047A, Takara, Japan). For miRNA detection, the miRNA First Strand cDNA Synthesis (Tailing Reaction) kit (B532451‐0020, Shanghai Sangon Biological Engineering Technology & Services, China) was used for reverse transcription to isolate the cDNA. The SYBR^®^ Premix EX Taq^TM^ II (Perfect Real Time) Kit (DRR081, Takara, Japan) and a real‐time fluorescence qPCR (ABI7500, Applied Biosystems, Foster City, CA, USA) were adopted for real‐time qPCR. The PCR amplification programme was set as a two‐step method, which was as follows: pre‐denaturation at 95°C for 30 seconds, followed by 40 PCR cycles (denaturation at 95°C for 5 seconds and annealing at 60°C for 34 seconds). Three replicate wells were set for each sample. The miRNA universal reverse primer and the forward primer of the internal reference U6 were provided in the miRNA First Strand cDNA Synthesis (Tailing Reaction) Kit, while the other primers were synthesized by Shanghai Sangon Biological Engineering Technology & Services (Shanghai, China; Table 2). The relative expression of the target was calculated based on the 2^−△△Ct^ method with β‐actin or U6 serving as internal control: △△Ct = △Ct_experimental group_^−^△Ct_control group_, △Ct = Ct (target gene)–Ct (internal reference).

**TABLE 2:** Primer sequences used for RT‐qPCR

Target gene	Primer sequences
LINC00472	F: 5'‐GATGGCAGCTGTCTCTCTCC‐3'
	R: 5'‐GGGCCTCTCTGACCGTATCT‐3'
ZEB1	F: 5'‐CTTAAGAGCGCTAGCTGCCA‐3'
	R: 5'‐CGCATTTTCTTTTTGGGCGG‐3'
miR‐23a‐3p	F: 5'‐ACACTCCAGCTGGGATCACATTGCCAGGGATTT‐3'
	R: 5'‐CTCAACTGGTGTCGTGGA‐3'
U6	F: 5'‐CTCGCTTCGGCAGCACA‐3'
	R: 5'‐AACGCTTCACGAATTTGCGT‐3'
β‐Actin	F: 5'‐CACTGTGCCCATCTACGAGG‐3'
	R: 5'‐TAATGTCACGCACGATTTCC‐3'
FOXO3	F: 5'‐TCACGCACCAATTCTAACGC‐3'
	R: 5'‐CACGGCTTGCTTACTGAAGG‐3'
BID	F: 5'‐AGGTGATTTAAGGGCCCAGGTC‐3'
	R: 5'‐CCGTGGCTTCATGTCTTTAGCA‐3'

Abbreviations: BID, BH3‐interacting domain death agonist; β‐actin, actin beta; F, forward; FOXO3, forkhead box O3; LINC00472, long intergenic non‐protein coding RNA 472; miR‐23a‐3p, microRNA‐23a‐3p; R, reverse; RT‐qPCR, reverse transcription‐quantitative polymerase chain reaction; U6, U6 small nuclear RNA; ZEB1, zinc finger E‐box binding homeobox 1.

### 2.7 Dual‐luciferase reporter assay

The potential binding sites of ZEB1 protein in LINC00472 promoter region and the binding site of FOXO3 in BID promoter region were identified through UCSC (http://genome.ucsc.edu/) and JASPAR (http://jaspar.genereg.net/). Recombinant luciferase reporter gene vectors with truncated or mutated binding site were constructed and cotransfected with oe‐ZEB1 and oe‐FOXO3 vectors into the HEK‐293T cells, respectively. Next, oe‐NC and oe‐ZEB1 were cotransfected with the LINC00472 promoter region 2 Kb luciferase reporter plasmid, respectively, to identify whether ZEB1 would bind to the LINC00472 promoter region. Subsequently, oe‐NC and oe‐FOXO3 were cotransfected with the BID promoter region 2 Kb luciferase reporter plasmid, respectively, to identify whether FOXO3 would bind to the BID promoter region. The cells were collected and lysed 48 hours after transfection, after which the luciferase activity was detected using a luciferase detection kit (K801‐200; BioVision, Mountain View, CA, USA) in a Glomax20/20 luminometer (Promega BioVision, Mountain View, CA, USA; Promega Corporation, Madison, WI, USA).

The wild‐type (WT) or mutant (MUT) sequence in the 3'‐untranslated region (3'UTR) of LINC00472 and FOXO3 was synthesized and cloned into the pMIR reporter in strict accordance with the provided instructions of the luciferase detection kit (Promega Corporation, Madison, WI, USA). The Lipofectamine 2000 reagent was adopted to cotransfect LINC00472 and FOXO3 WT or MUT 3'UTR and miR‐23a‐3p mimic or mimic NC into the HEK‐293T cells. The cells were harvested for detection of the luciferase activity using the dual‐luciferase reporter system (Promega Corporation, Madison, WI, USA) 48 hours after transfection. Each experiment was conducted three times independently.

### 2.8 Cell counting kit‐8 (CCK‐8) assay

Cell viability was evaluated by CCK‐8 assay (CK04, Dojindo, Japan). After transfection, the pancreatic cancer cells were resuspended and seeded into 96‐well plates at a density of 2 × 10^3^ cells with 100 µL per well. The cells were then periodically incubated for 0, 24, 48, 72 and 96 hours. CCK‐8 assay was conducted at the aforementioned time‐points with the addition of 10 µL of CCK‐8 solution into each well. Following incubation for 2 hours, the optical density (OD) value of each well was detected using a microplate reader at the excitation wavelength of 450 nm.

### 2.9 Western blot analysis

The cells were harvested by trypsin detachment and lysed with the enhanced radioimmunoprecipitation assay lysis buffer (BOSTER, Wuhan, China) containing several protease inhibitors, after which the protein concentration was determined using the bicinchoninic acid kit (BOSTER, Wuhan, China). The protein content was separated by 10% sodium dodecyl sulphate‐polyacrylamide gel electrophoresis, and then transferred onto a polyvinylidene fluoride membrane. The membrane was incubated with 5% bovine serum albumin at room temperature for 2 hours to terminate any non‐specific binding and then incubated at 4°C overnight with the following diluted primary antibodies: rabbit polyclonal to caspase‐3 (ab13847, at a dilution ratio 1:500), rabbit polyclonal to cleaved caspase‐3 (ab49822, at a dilution ratio 1:500), rabbit monoclonal [E63] to Bcl‐2–associated X protein (Bax) (ab32503, at a dilution ratio 1:1000), rabbit monoclonal [SP6] to Ki‐67 (ab16667, at a dilution ratio 1:500), rabbit polyclonal to proliferating cell nuclear antigen (PCNA) (ab18197, at a dilution ratio 1:1000), rabbit monoclonal [Y8] to Bid (ab32060, at a dilution ratio 1:1000), rabbit polyclonal to beta actin (ab8227, at a dilution ratio 1:200) and rabbit polyclonal to FOXO3A‐ChIP grade (ab12162, at a dilution ratio 1:2500) (all from Abcam, Cambridge, UK). The following day, the membrane was further incubated with the diluted horseradish peroxidase‐labelled goat anti‐rabbit IgG (ab205719, at a dilution ratio 1:2000, Abcam BioVision, Mountain View, CA, USA; Promega Corporation, Madison, WI, USA) at room temperature for 1 hour. The immunoblots were visualized using the enhanced chemiluminescence reagent (EMD Millipore, Billerica, MA, USA). ImageJ software (Bio‐Rad, Hercules, CA, USA) was adopted to quantify the grey value of target protein where β‐actin was used as internal reference. Each experiment was conducted 3 times independently.

### 2.10 Flow cytometry

Annexin V‐fluorescein isothiocyanate (FITC)/propidium iodide (PI) double staining was conducted to evaluate the degree of cell apoptosis. After 48 hours of transfection, the cell concentration was adjusted to 1 × 10^6^ cells/mL. The cells were fixed using 70% precooled ethanol overnight at 4°C, after which 100 μL of the cell suspension (no less than 10^6^ cells/mL) was resuspended in 200 μL of binding buffer. Subsequently, the cells were stained with 10 μL Annexin V‐FITC and 5 μL PI for 15 minutes at room temperature in conditions devoid of light. After the addition of 300 μL of binding buffer, the degree of cell apoptosis was determined by flow cytometry at the excitation wavelength of 488 nm. A flow cytometer was employed to analyse 2 × 10^4^ cells per repetition.

### 2.11 RNA pull‐down assay

The cells were transfected with biotinylated WT miR‐23a‐3p and MUT miR‐23a‐3p (50 nmol L^−1^ each). Forty‐eight hours later, the cells were isolated and rinsed with PBS, followed by incubation with the specific lysis buffer (Ambion, Austin, TX, USA) for 10 minutes. The lysate was incubated with the M‐280 streptavidin magnetic beads (Sigma‐Aldrich, St. Louis, MO, USA) pre‐coated with RNase‐free and yeast tRNA (Sigma‐Aldrich, St. Louis, MO, USA) at 4°C for 3 hours, and then rinsed twice with cold lysis buffer. RT‐qPCR was conducted to determine the expression patterns of LINC00472 and FOXO3.

### 2.12 Chromatin immunoprecipitation (ChIP)

The ChIP kit (Millipore, Billerica, MA, USA) was used for this assay. Upon attaining 70%‐80% cell confluence, the cells were fixed in 1% formaldehyde at room temperature for 10 minutes to produce DNA‐protein cross‐linking. The cells were then subjected to ultrasonic treatment to produce chromatin fragments of appropriate size at 120 w, 2‐s on and 5‐s off each time, for a total of 15 cycles. Subsequently, the cells were subject to centrifugation at 28341.3 *g* at 4°C after which the supernatant was collected and divided into three tubes, which were incubated with the positive control antibody to RNA polymerase II, NC antibody to normal human IgG and rabbit anti‐FOXO3 (at a dilution ratio 1:100, ab12162, Abcam, Cambridge, UK) overnight at 4°C. Protein Agarose/Sepharose was added for precipitation of the endogenous DNA‐protein complex. After centrifugation and removal of the non‐specific complex, the DNA‐protein complex was subject to overnight incubation at 65°C to terminate cross‐linking. Phenol/chloroform was then added to extract and purify the isolated DNA fragments. Subsequently, RT‐qPCR was conducted to determine the expression pattern of BID promoter.^20^

### 2.13 Tumour xenografts in vivo

A total of 108 healthy male nude mice aged 6‐8 weeks (Beijing Institute of Pharmacology, Chinese Academy of Medical Sciences, Beijing, China) were separately housed in a specific‐pathogen‐free animal laboratory. The laboratory conditions were 22‐25°C under 60%‐65% relative humidity with a 12‐hour light/dark cycle with ad libitum access to food and water. The experiment was started after 1 week of acclimation. The health conditions of the nude mice were observed before the experiment. The cells were stably infected with the lentivirus expressing oe‐NC, oe‐LINC00472, sh‐NC, sh‐LINC00472, oe‐FOXO3 or in combination. Approximately a concentration of 2 × 10^6^ cells were suspended in 200 μL of PBS and then injected subcutaneously into the left or right hind legs of nude mice. The diameter of the transplanted tumour was documented on a weekly basis, and the tumour volume was calculated to plot the tumour growth curve using the following formula: tumour volume = (a * b^2^)/2 (a is the longest diameter of the tumour; b is the shortest diameter of the tumour). After 28 days, the nude mice were killed, and the tumours were weighed and measured. The tumours were subsequently used for experimental procedures such as RT‐qPCR, Western blot analysis, immunohistochemistry and terminal deoxynucleotidyl transferase–mediated dUTP‐biotin nick end labelling (TUNEL) staining.

### 2.14 Immunohistochemistry

The paraffin‐embedded tumour tissue sections were taken for immunohistochemical analysis. The sections were dewaxed in water and dehydrated with gradient ethanol solutions. The sections were rinsed under tap water for 2 minutes, 3% methanol in H_2_O_2_ for 20 minutes, distilled water for 2 minutes and 0.1 mol L^−1^ PBS for 3 minutes. After antigen retrieval in water bath, the sections were cooled under tap water. Normal goat serum (C‐0005, Shanghai Haoran Biotechnology Co., Ltd., Shanghai, China) was added in a dropwise manner onto the sections and incubated at room temperature for 20 minutes. The sections were incubated with the primary antibody, rabbit monoclonal [SP6] to Ki‐67 (ab16667, at a dilution ratio 1:500, Abcam, Cambridge, UK) overnight at 4°C. Next, the sections were rinsed with 0.1 mol L^−1^ PBS for 3 times (5 min/time). The secondary antibody, goat anti‐rabbit IgG, was added in a dropwise manner onto the sections and incubated at 37°C for 20 minutes, followed by 3 rinses. The sections were incubated with the horseradish‐labelled working solution (0343‐10000U, Imunbio Co., Ltd., Beijing, China) at 37°C for 20 min, and then rinsed with 0.1 mol L^−1^ PBS for 3 times, each time for 5 minutes. Thereafter, the sections were stained with diaminobenzidine (ST033; Guangzhou Weijia Technology Co., Ltd., Guangzhou, China) and counter‐stained with haematoxylin (PT001; Shanghai Bogu Biotechnology Co., Ltd., Shanghai, China) for 1 minute. The sections were stained blue with 1% ammonia in water, rinsed with water, dehydrated with gradient ethanol solutions, cleared with xylene and mounted by neutral gum. Next, the sections were observed under a microscope. In each section, five high‐power fields were randomly selected with counting of 100 cells in each field. Positive cells <10% were defined as negative, 10% ≤positive cells <50% were defined as positive, and positive cells >50% were defined as strong positive.

### 2.15 TUNEL assay

Tumour tissues were fixed with 4% paraformaldehyde, dehydrated by ascending degrees of alcohol, cleared using xylene, embedded in paraffin and cut into 5‐µm sections. The tissue sections were deparaffinized and proceeded in strict accordance with the conventional histological protocols. To terminate any peroxidase activity, the tissue sections were immersed in 3% hydrogen peroxide solution in ethanol for 15 minutes. After a rinse, the tissue sections were incubated with proteinase K for 20 minute at room temperature. After a rinse, the tissue sections were incubated with the reaction solution provided with the TUNEL staining kit. After another rinse, the tissue sections were incubated with diaminobenzidine for 15 minutes at room temperature and then stained using haematoxylin following another rinse. Lastly, the cells with brown nucleus were regarded as TUNEL‐positive cells.

### 2.16 Statistical analysis

All experimental data were analysed using SPSS 21.0 software (IBM Corp. Armonk, NY, USA). Measurement data were summarized by mean ± standard deviation from at least three independent experiments. Comparison between two groups was performed by the unpaired *t* test. Comparison among multiple groups was performed by one‐way analysis of variance (ANOVA) with Tukey's post hoc test. Comparison among groups at different time‐points was performed using two‐way ANOVA or repeated‐measures ANOVA with Bonferroni's post hoc test. Pearson's correlation analysis was adopted to analyse the correlation between ZEB1 and LINC00472, miR‐23a‐3p and LINC00472, and FOXO3 and BID. The Kaplan‐Meier method was adopted to analyse the survival data, and the log‐rank test was conducted for comparison between different survival curves. In all statistical references, a value of *P* < 0.05 was indicative of a statistically significant difference.

## 3 RESULTS

### 3.1 LINC00472 expression was inhibited by zeb1 in pancreatic cancer

**FIGURE 1:**
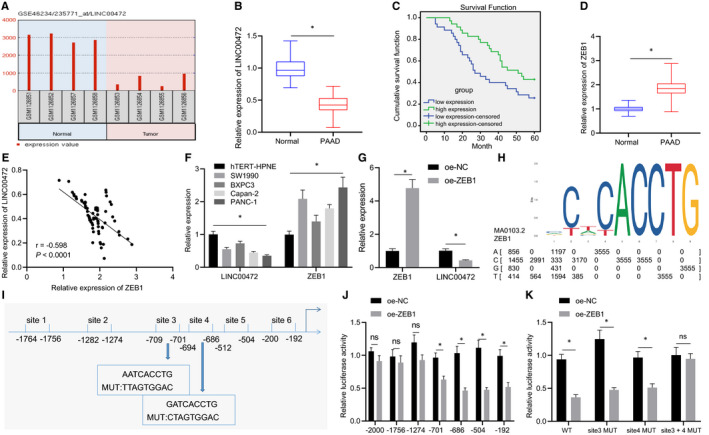
Expression pattern of LINC00472 was suppressed by ZEB1 in pancreatic cancer. A, GEO2R analysis (https://www.ncbi.nlm.nih.gov/geo/geo2r/) of LINC00472 expression pattern in pancreatic cancer in the GSE46234 data set. B, RT‐qPCR determination of LINC00472 expression pattern in 70 pairs of pancreatic cancer tissues and adjacent normal tissues. C, The Kaplan‐Meier curve of patient's overall survival. D, RT‐qPCR determination of ZEB1 expression pattern in 70 pairs of pancreatic cancer tissues and adjacent normal tissues. E, Analysis of the correlation between ZEB1 expression pattern and LINC00472 expression pattern in the pancreatic cancer tissues. F, RT‐qPCR determination of LINC00472 and ZEB1 in hTERT‐HPNE, SW1990, BXPC3, Capan‐2 and PANC‐1 cells. G, The expression patterns of ZEB1 and LINC00472 were determined by RT‐qPCR. H, Sequence logo of ZEB1 binding site. I, JASPAR website predicted the binding site between ZEB1 and the promoter region of LINC00472 (−2000, +50). J, Luciferase activity was detected dual‐luciferase reporter assay. K, Luciferase activity was detected. Data between the cancer tissues and adjacent normal tissues were compared by the paired *t* test. Data between the other two groups were compared by the unpaired *t* test. Data among multiple groups were compared by one‐way ANOVA with Tukey's post hoc test. The Kaplan‐Meier method was adopted to calculate the overall survival of patients, and the survival difference of patients was analysed by log‐rank analysis. * *P* < 0.05

Existing literature has demonstrated the functionality of LINC00472 as a tumour suppressor in various type of cancers including lung cancer,^9^ colorectal cancer^10^ and ovarian epithelial cancer^21^; however, its role in pancreatic cancer remains unidentified. We initially analysed the GSE46234 data set by GEO2R (https://www.ncbi.nlm.nih.gov/geo/geo2r/) and identified that LINC00472 was poorly expressed in pancreatic cancer (Figure 1A), which was further tested and verified by an array of experiments in the clinical patient tissues.

The expression pattern of LINC00472 in the pancreatic cancer tissues and adjacent normal tissues was determined by RT‐qPCR. The results showed that the expression pattern of LINC00472 in pancreatic cancer tissues was reduced compared with the adjacent normal tissues (Figure 1B). Using an analysis of the relationship between LINC00472 expression and the clinicopathological characteristics of the patients, we identified explicit correlations between the LINC00472 expression pattern and the patient's TNM stage and lymph node metastasis but not with gender and age (Table 3), and we further observed an inferior overall survival of patients with low expression (Figure 1C).

**TABLE 3:** Relationship between LINC00472 expression and clinicopathological characteristics of patients with pancreatic cancer

Clinicopathological characteristics	LINC00472 expression		*X^2^*	*P*
	High (n = 35)	Low (n = 35)		
Age (y)				
≥60	16	17	0.057	0.811
<60	19	18		
Gender				
Male	22	20	0.238	0.626
Female	13	15		
TNM stage				
I‐II	22	12	5.719	0.017
III‐IV	13	23		
Lymph node metastasis				
Negative	22	9	7.124	0.008
Positive	15	26		

Abbreviations: LINC00472, long intergenic non‐protein coding RNA 472; TNM, tumour, node, metastasis.

The expression pattern of LINC00472 was down‐regulated in pancreatic cancer, but the relative mechanism has not been determined yet. As an epithelial‐mesenchymal transition (EMT) marker, ZEB1 could evidently promote the development of pancreatic cancer^16^, ^22^ and it functioned as a transcription factor to suppress the expression pattern of its corresponding downstream target genes.^23^ In order to determine whether ZEB1 can regulate the expression pattern of LINC00472 in the progression of pancreatic cancer, we further examined the expression pattern of ZEB1 in the pancreatic cancer tissues and adjacent normal tissues by RT‐qPCR. The results displayed that, compared with the adjacent normal tissues, the expression pattern of ZEB1 was increased in the pancreatic cancer tissues (Figure 1D), while a negative correlation was depicted between the ZEB1 expression pattern and the expression pattern of LINC00472 (Figure 1E). Concurrently, RT‐qPCR was conducted to determine the expression patterns of LINC00472 and ZEB1 in the normal immortalized human pancreatic epithelial cell line hTERT‐HPNE and four human pancreatic cancer cell lines SW1990, BXPC3, Capan‐2 and PANC‐1, the results of which demonstrated that the expression pattern of LINC00472 was lower while the expression pattern of ZEB1 was significantly higher in the pancreatic cancer cells compared with the hTERT‐HPNE cells (Figure 1F). The BXPC3 and PANC‐1 cell lines with large fold change in LINC00472 and ZEB1 expression were selected for subsequent experimentation. After overexpressing ZEB1 in the BXPC3 cells, the expression pattern of LINC00472 was reduced, thus suggesting that ZEB1 may negatively regulate the expression pattern of LINC00472 (Figure 1G). The JASPAR website predicted that ZEB1 might bind to the promoter region of the LINC00472 gene (−2000, +50) (Figure 1H,I), which was further verified by dual‐luciferase reporter assay. Our findings revealed that, after truncating site 3 of the promoter region of LINC00472 gene, overexpression of ZEB1 decreased the luciferase activity, while truncating of site 4 further reduced the luciferase activity (Figure 1J). Additionally, the luciferase activity had decreased after mutating site 3 or site 4 separately, while after simultaneous mutation of the site 3 and site 4, the overexpression of ZEB1 did not affect the luciferase activity (Figure 1K). The aforementioned data suggested that site 3 and site 4 were involved in the regulation of LINC00472 expression pattern by ZEB1.

### 3.2 LINC00472 inhibited proliferation of pancreatic cancer cells while promoting their apoptosis

**FIGURE 2:**
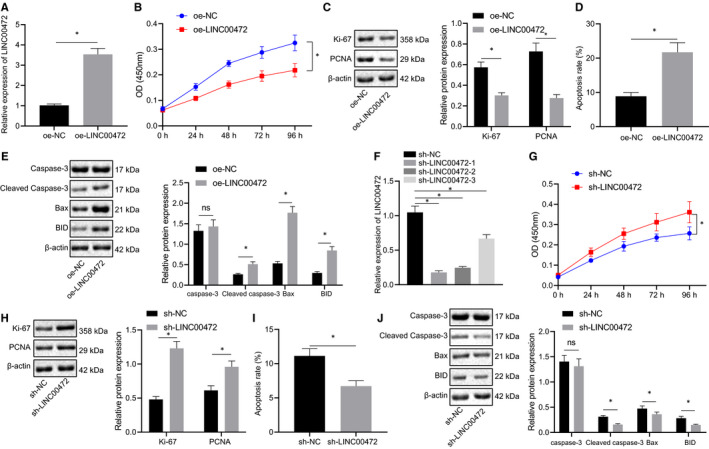
LINC00472 impeded pancreatic cancer cell proliferation and promoted their apoptosis. A, RT‐qPCR determination of LINC00472 expression. B, CCK‐8 showing proliferation of PANC‐1 cells transfected with oe‐LINC00472. C, Western blots of Ki‐67 and PCNA proteins in PANC‐1 cells transfected with oe‐LINC00472. D, Flow cytometric monitoring of apoptosis of PANC‐1 cells transfected with oe‐LINC00472. E, Western blots of caspase‐3, cleaved caspase‐3, BID and Bax proteins in PANC‐1 cells transfected with oe‐LINC00472. F, RT‐qPCR determination of LINC00472 expression pattern. G, CCK‐8 showing proliferation of BXPC3 cells transfected with sh‐LINC00472. H, Western blots of Ki‐67 and PCNA proteins in BXPC3 cells transfected with sh‐LINC00472. I, Flow cytometric monitoring of apoptosis of BXPC3 cells transfected with sh‐LINC00472. J, Western blots of caspase‐3, cleaved caspase‐3, BID, and Bax proteins in BXPC3 cells transfected with sh‐LINC00472. Data between two groups were compared by the unpaired *t* test. Data among multiple groups were compared by one‐way ANOVA with Tukey's post hoc test. * *P* < 0.05

To further examine the effects of LINC00472 on the pancreatic cancer cells, LINC00472 was first overexpressed in the PANC‐1 cells, after which RT‐qPCR validated the high overexpression efficiency of LINC00472 (Figure 2A). Subsequently, the degree of cell proliferation and apoptosis was assayed by CCK‐8 experiment and flow cytometry, respectively, while the expression of the proliferation‐related factors (Ki‐67 and PCNA) and apoptosis‐related proteins (cleaved caspase‐3, BID and Bax) was examined by Western blot analysis. Our results revealed that cell proliferation was suppressed (Figure 2B), while cell apoptosis was enhanced upon LINC00472 overexpression (Figure 2D). Correspondingly, the expression patterns of Ki‐67 and PCNA were reduced (Figure 2C), while the expression patterns of cleaved caspase‐3, BID and Bax were increased after overexpression of LINC00472 (Figure 2E).

LINC00472 was knocked down by shRNA in the BXPC3 cells, and its efficiency was determined by RT‐qPCR. The sh‐LINC00472‐1 exhibiting superior silencing efficiency was chosen for subsequent cell experimentation (Figure 2F). The results of CCK‐8, flow cytometry and Western blot analysis demonstrated an enhancement in cell proliferation (Figure 2G), a suppression in cell apoptosis (Figure 2I), elevations in Ki‐67 and PCNA protein levels (Figure 2H), and reductions in cleaved caspase‐3, BID and Bax protein levels (Figure 2J) upon LINC00472 knockdown. In summary, the aforementioned data demonstrated that LINC00472 had successfully exerted antiproliferative and pro‐apoptotic effects on the pancreatic cancer cells.

### 3.3 LINC00472 inhibited tumorigenicity of pancreatic cancer cells in vivo

**FIGURE 3:**
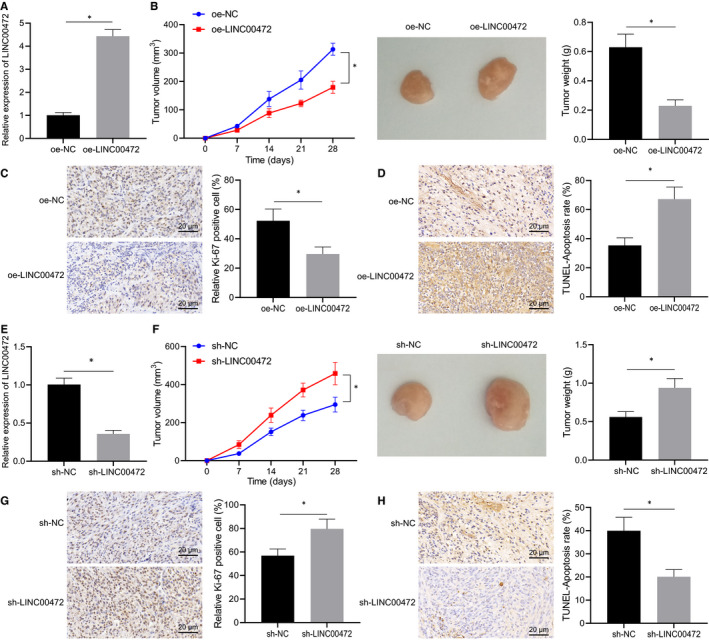
LINC00472 suppressed tumorigenesis in vivo. A, RT‐qPCR determination of the expression pattern of LINC00472. B, Growth curve, photographs and weight statistics of tumours in nude mice injected with PANC‐1 overexpressing LINC00472. C, IHC detection of Ki‐67 expression pattern in tumour tissues in nude mice injected with PANC‐1 overexpressing LINC00472. D, TUNEL staining of cell apoptosis in tumour tissues in nude mice injected with PANC‐1 overexpressing LINC00472. E, RT‐qPCR determination of the expression pattern of LINC00472. F, Growth curve, photographs and weight statistics of tumours in nude mice injected with PANC‐1 overexpressing LINC00472. G, IHC detection of Ki‐67 expression pattern in the tumour tissues of nude mice injected with BXPC3 infected with lentivirus expressing sh‐LINC00472. H, TUNEL staining of cell apoptosis in tumour tissues of nude mice injected with BXPC3 infected with lentivirus expressing sh‐LINC00472, n = 12/group. Data between two groups were compared by the unpaired *t* test. Comparison among groups at different time‐points was performed using repeated‐measures ANOVA with Bonferroni's post hoc test. * *P* < 0.05

The stable infected cell line PANC‐1 overexpressing LINC00472 was constructed and subcutaneously injected into the nude mice to validate the effect of LINC00472 in vivo. RT‐qPCR was conducted to determine the expression pattern of LINC00472 in the tumour tissues, the results of which confirmed the up‐regulation of LINC00472 in the nude mice subject to injection with oe‐LINC00472–treated cells (Figure 3A). Tumour growth curves, photographs of tumours and weight statistics demonstrated that overexpression of LINC00472 inhibited tumorigenesis in vivo (Figure 3B). The expression pattern of Ki‐67 in the tumours was subsequently detected by IHC, and the results displayed that overexpression of LINC00472 had reduced the expression pattern of Ki‐67 (Figure 3C). Meanwhile, TUNEL staining results verified that overexpression of LINC00472 induced cell apoptosis (Figure 3D).

The established stable cell line BXPC3 infected with lentivirus expressing sh‐LINC00472 was subcutaneously injected for tumorigenesis. RT‐qPCR successfully ascertained the LINC00472 knockdown in the nude mice (Figure 3E). Consequently, LINC00472 silencing resulted in facilitated tumorigenicity of pancreatic cancer cells in vivo (Figure 3F), reduced expression pattern of Ki‐67 (Figure 3G) and suppressed cell apoptosis (Figure 3H). Conjointly, these results demonstrated that LINC00472 inhibited the tumorigenesis of pancreatic cancer cells in vivo.

### 3.4 LINC00472 competitively bound to miR‐23a‐3p to up‐regulate foxo3 expression

**FIGURE 4:**
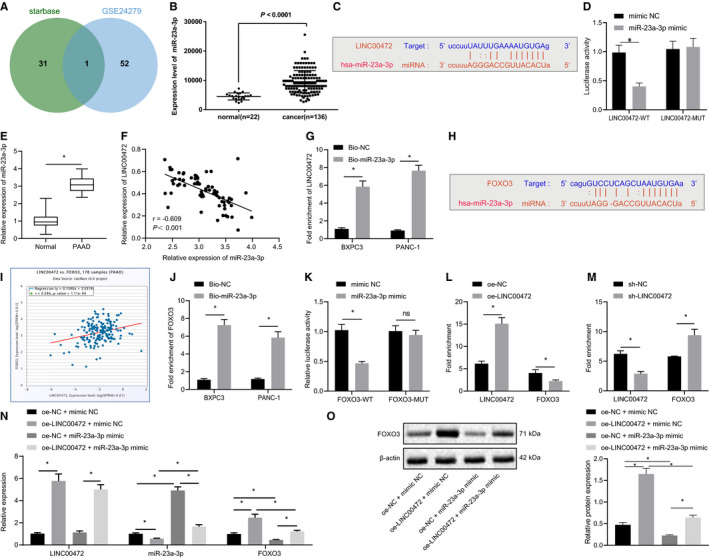
LINC00472 promotes the expression of FOXO3 through competitive binding to miR‐23a‐3p. A, Venn diagram of miRNAs binding to LINC00472 predicted by the starBase website and up‐regulated miRNAs in the GSE24279 data set. B, miR‐23a‐3p expression pattern in the normal and tumour samples in the GSE24279 data set. C, The binding sites between LINC00472 and miR‐23a‐3p predicted by the starBase website. D, The binding between LINC00472 and miR‐23a‐3p verified by dual‐luciferase reporter assay. E, RT‐qPCR determination of the expression pattern of miR‐23a‐3p in 70 pancreatic cancer tissues and adjacent normal tissues. F, Correlation analysis of LINC00472 expression pattern and miR‐23a‐3p expression pattern in the pancreatic cancer tissues. G, RNA pull‐down assay was used to verify the binding between LINC00472 and miR‐23a‐3p. H, The binding site between miR‐23a‐3p and FOXO3 3'UTR predicted by starBase website. I, Correlation analysis of LINC00472 expression and FOXO3 expression analysed by the starBase website. J, RNA pull‐down assay was used to verify the binding between miR‐23a‐3p and FOXO3. K, Dual‐luciferase reporter gene assay was used to confirm the binding between miR‐23a‐3p and FOXO3 3'UTR. L, After overexpressing LINC00472 in the pancreatic cancer cell PANC‐1, RNA pull‐down was used to detect changes in miR‐23a‐3p binding to LINC00472 or FOXO3. M, After silencing LINC00472 in pancreatic cancer cell BXPC3, RNA pull‐down was used to detect changes in miR‐23a‐3p binding to LINC00472 or FOXO3. N, The expression patterns of LINC00472, miR‐23a‐3p and FOXO3 were determined by RT‐qPCR. O, Western blots of LINC00472, miR‐23a‐3p and FOXO3. Data between two groups were compared by the unpaired *t* test. Data among multiple groups were compared by one‐way ANOVA with Tukey's post hoc test. Pearson's correlation analysis was used to analyse the correlation between LINC00472 and miR‐23a‐3p. * *P* < 0.05

Accumulating evidence has elicited that LINC00472 can function as a ceRNA to regulate the occurrence and development of various diseases.^9^, ^10^ In the current study, in order to further examine the regulatory mechanism of LINC00472 in pancreatic cancer, the starBase website (http://starbase.sysu.edu.cn/) was used to predict miRNAs that may bind to LINC00472. The obtained miRNAs were intersected with the up‐regulated miRNAs in the GSE24279 data set, the results of which identified that miR‐23a‐3p could independently bind to LINC00472 (Figure 4A), and moreover, miR‐23a‐3p was highly expressed in pancreatic cancer (Figure 4B). The specific binding sites between eLINC00472 and miR‐23a‐3p are shown in Figure 4C. The results of dual‐luciferase reporter assay indicated that miR‐23a‐3p mimic inhibited the luciferase activity of LINC00472‐WT without altering that of LINC00472‐MUT (Figure 4D). Meanwhile, a high expression pattern of miR‐23a‐3p was reported in pancreatic cancer.^12^ Therefore, we speculated that LINC00472 may regulate the development of pancreatic cancer through miR‐23a‐3p. Furthermore, the expression pattern of miR‐23a‐3p in the 70 pairs of pancreatic cancer tissues and adjacent normal tissues was determined by RT‐qPCR. The results showed that miR‐23a‐3p was up‐regulated in the pancreatic cancer tissues (Figure 4E), while the miR‐23a‐3p expression pattern was negatively correlated with the LINC00472 expression pattern (Figure 4F). Besides, the results of RNA pull‐down experiments demonstrated that the biotin‐labelled miR‐23a‐3p probe can disintegrate LINC00472, thus verifying that LINC00472 could bind to miR‐23a‐3p (Figure 4G).

Then, we predicted the potential target genes of miR‐23a‐3p through starBase and identified that miR‐23a‐3p could bind to FOXO3 3’UTR (Figure 4H). A positive correlation was identified between LINC00472 and FOXO3 in pancreatic cancer (Figure 4I). The results of RNA pull‐down experiments demonstrated that the biotin‐labelled miR‐23a‐3p probe can disintegrate the FOXO3 mRNA, demonstrating that miR‐23a‐3p could bind to FOXO3 mRNA (Figure 4J), and the dual‐luciferase reporter gene assay revealed that miR‐23a‐3p mimic inhibited the luciferase activity of WT‐FOXO3, while it had a minor effect on mutated FOXO3 (Figure 4K). The results of RNA pull‐down assay revealed that the binding between miR‐23a‐3p and LINC00472 was enhanced after LINC00472 overexpression, while the binding between miR‐23a‐3p and FOXO3 was attenuated (Figure 4L). Conversely, such alterations can be neutralized by silencing LINC00472 (Figure 4M). Finally, an increased expression pattern of FOXO3 was identified after overexpressing LINC00472 in the PANC‐1 cells, while the expression pattern of FOXO3 was reduced after overexpressing miR‐23a‐3p. However, simultaneous overexpression of miR‐23a‐3p could annul the regulatory effects on FOXO3 expression pattern induced by overexpressing LINC00472, while LINC00472 could neutralize the weakening effects on FOXO3 expression caused by overexpressing miR‐23a‐3p separately (Figure 4N‐O). To summarize, the preceding results indicated that LINC00472 increased the FOXO3 expression by competitively binding to miR‐23a‐3p.

### 3.5 LINC00472 silencing enhanced the proliferation and impeded apoptosis of pancreatic cancer cells by down‐regulating FOXO3

**FIGURE 5:**
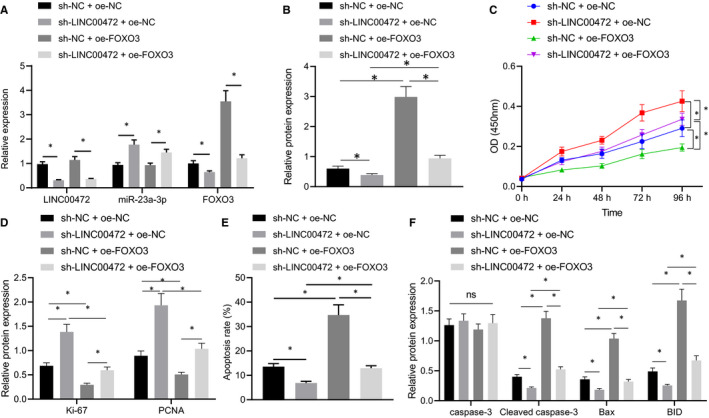
LINC00472 silencing boosted the proliferation and repressed the apoptosis of pancreatic cancer cells by inhibiting FOXO3. A, RT‐qPCR determination of the expression patterns of LINC00472, miR‐23a‐3p and FOXO3. B, Western blots of FOXO3 protein in the BXPC3 cells. C, CCK‐8 examined cell proliferation of BXPC3 cells. D, Western blots of Ki‐67 and PCNA proteins in the BXPC3 cells. E, Flow cytometry monitored the apoptosis of BXPC3 cells. F, Western blots of caspase‐3, cleaved caspase‐3, BID and Bax proteins in BXPC3 cells. Data between two groups were compared by the unpaired *t* test. Data among multiple groups were compared by one‐way ANOVA with Tukey's post hoc test. * *P* < 0.05

To investigate whether LINC00472 could mediate FOXO3 to influence pancreatic cancer cell functions, LINC00472 was silenced in the pancreatic cancer cell BXPC3 overexpressing FOXO3, after which RT‐qPCR was conducted to determine the expression patterns of LINC00472, miR‐23a‐3p and FOXO3, while Western blot analysis was conducted to determine the protein expression pattern of FOXO3. The expression pattern of FOXO3 was decreased, while the expression pattern of miR‐23a‐3p was increased upon individual LINC00472 silencing. The expression pattern of FOXO3 reduced by LINC00472 silencing could be annulled by oe‐FOXO3 transfection (Figure 5A,B). Correspondingly, LINC00472 silencing could facilitate cell proliferation, suppress cell apoptosis, elevate the expression patterns of proliferation markers Ki‐67 and PCNA and reduce the expression pattern of apoptosis markers cleaved caspase‐3, BID and Bax. However, overexpressing FOXO3 resulted in suppression of the proliferation but improvement in cell apoptosis. Moreover, overexpressing FOXO3 could neutralize the regulatory effects of LINC00472 silencing on cell proliferation and apoptosis (Figure 5C‐F). The preceding results indicated that LINC00472 silencing stimulated the proliferation of pancreatic cancer cells and reduced apoptosis by down‐regulating FOXO3.

### 3.6 LINC00472 knockdown enhanced tumorigenicity of pancreatic cancer cells through down‐regulating FOXO3

**FIGURE 6:**
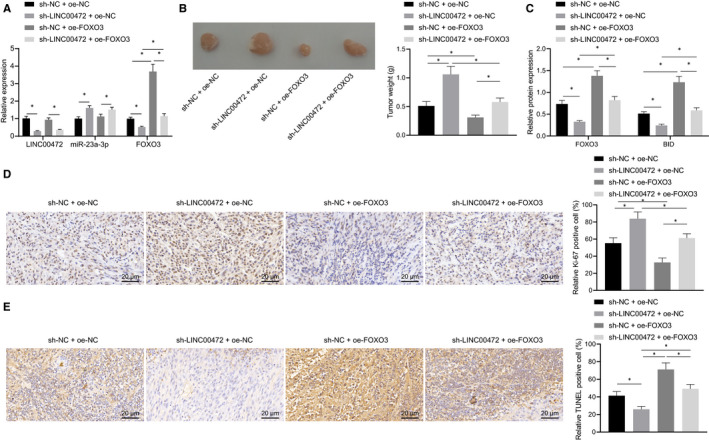
Silencing of LINC00472 promoted tumorigenesis in vivo through suppressing FOXO3. A, RT‐qPCR determination of the expression patterns of LINC00472, miR‐23a‐3p and FOXO3. B, Photographs and weight statistics of tumours. C, Western blots of FOXO3 and BID proteins. D, IHC detection of Ki‐67 expression pattern in the tumour tissues. E, TUNEL staining of cell apoptosis in tumour tissues. n = 12/group. Data between two groups were compared by the unpaired *t* test. Data among multiple groups were compared by one‐way ANOVA with Tukey's post hoc test. * *P* < 0.05

To further verify the effects of LINC00472 and FOXO3 on the pancreatic cancer cells in vivo, we subcutaneously injected the BXPC3 cells into nude mice to establish a tumorigenic model in vivo. The expression patterns of LINC00472, miR‐23a‐3p and FOXO3 in the tumour tissues were determined by means of RT‐qPCR, the results of which showed that the expression pattern of FOXO3 was reduced, while the expression of miR‐23a‐3p was elevated in response to successful lentivirus‐mediated LINC00472 silencing, and while the FOXO3 expression was neutralized by lentivirus‐packaged oe‐FOXO3 (Figure 6A). Our findings demonstrated that the tumour growth had improved in response to LINC00472 silencing, while the tumour growth was inhibited after FOXO3 overexpression. Moreover, overexpressing FOXO3 could reverse the stimulative effects on the pancreatic cancer cells mediated by LINC00472 silencing (Figure 6B). Concurrently, Western blot analysis of the FOXO3 and BID proteins elicited that the BID protein expression pattern was decreased after LINC00472 silencing but elevated by FOXO3 overexpression, whereas restoration of FOXO3 could obliterate the inhibitory effects of LINC00472 silencing on BID (Figure 6C). Finally, the expression pattern of Ki‐67 in the tumour tissues was detected by IHC, while the degree of apoptosis of the tumour cells was assessed by TUNEL staining. The results demonstrated that the expression pattern of Ki‐67 was increased while the apoptosis was suppressed after individually silencing LINC00472; however, overexpression of FOXO3 individually resulted in conflicting effects. Furthermore, overexpressing FOXO3 could annul the regulatory effects mediated by LINC00472 silencing on Ki‐67 expression pattern and cell apoptosis (Figure 6D,E). Altogether, the preceding results indicated that LINC00472 knockdown amplified the tumorigenic capacity of pancreatic cancer cells by suppression of FOXO3.

### 3.7 FOXO3 transcriptionally activated BID expression

**FIGURE 7:**
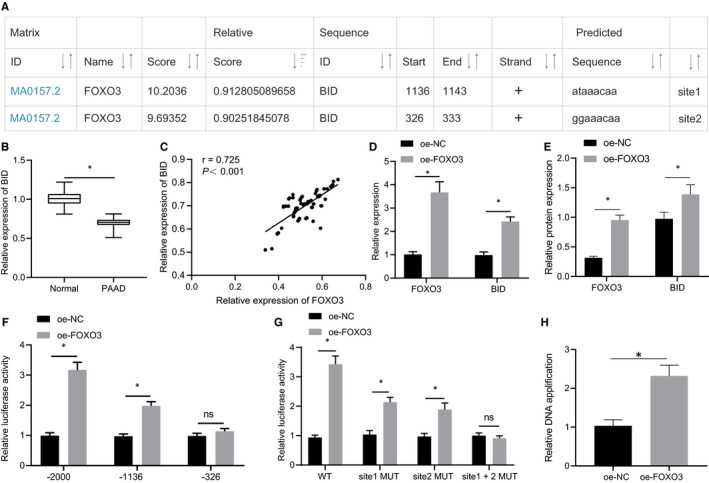
FOXO3 activated the expression pattern of BID. A, JASPAR website prediction of the binding site between FOXO3 and the promoter region of BID. B, RT‐qPCR determination of the expression pattern of BID in 70 pairs of pancreatic cancer tissues and adjacent normal tissues. C, Analysis of the correlation between the FOXO3 expression pattern and BID expression pattern in the pancreatic cancer tissues. D, RT‐qPCR determination of the expression pattern of FOXO3 and BID. E, Western blots of FOXO3 and BID proteins. F, G, Dual‐luciferase reporter gene assay was performed to assess that binding between FOXO3 and the promoter region of BID. H, Enrichment of FOXO3 in the BID promoter region determined by ChIP assay. Data between two groups were compared by the unpaired *t* test. Pearson's correlation analysis was used to analyse the correlation between FOXO3 and BID. * *P* < 0.05

As a transcription factor, FOXO3 can initiate the expression patterns of its target genes.^24^ The the JASPAR website predicted the unidentified binding sites of FOXO3 in the promoter region of the BID gene (Figure 7A). As determined by RT‐qPCR, the expression pattern of BID was lower in the pancreatic cancer tissues relative to the adjacent normal tissues (Figure 7B), and it was positively correlated with the FOXO3 expression pattern (Figure 7C). Therefore, we speculated that LINC00472 may regulate the expression pattern of BID through FOXO3 to subsequently manipulate the development of pancreatic cancer.

After overexpressing FOXO3 in the PANC‐1 cells, we determined elevations in the BID mRNA and protein levels by RT‐qPCR and Western blot analysis (Figure 7D,E). The BID gene promoter region was further truncated, and the results of dual‐luciferase reporter gene assay showed that compared with the oe‐NC‐treated cells, after truncating site 1, overexpression of FOXO3 increased the luciferase activity, while after truncating both site 1 and site 2, the BID luciferase activity was unaffected by overexpression of FOXO3 (Figure 7F). Additionally, compared with the oe‐NC–treated cells, after mutating site 1 or site 2 individually, overexpression of FOXO3 increased the luciferase activity. However, after simultaneous mutation of both site 1 and site 2, the BID luciferase activity was unaffected after overexpression of FOXO3 (Figure 7G), indicating that FOXO3 could regulate BID gene via site 1 and site 2. Moreover, the detection results of the ChIP assay showed that the BID promoter signal was enhanced after FOXO3 overexpression (Figure 7H). The aforementioned evidence indicated that FOXO3 activated the expression of BID through transcription.

### 3.8 Silencing of LINC00472 inhibited BID expression through miR‐23a‐3p/FOXO3 to regulate pancreatic cancer cell proliferation and apoptosis

**FIGURE 8:**
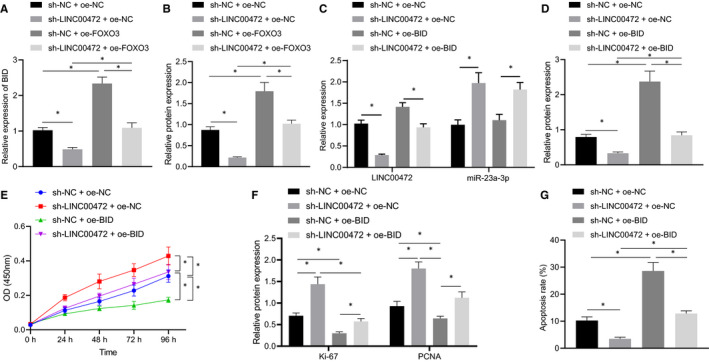
Silencing of LINC00472 suppressed BID expression through miR‐23a‐3p/FOXO3 to promote proliferation and inhibit apoptosis of pancreatic cancer cells. A, RT‐qPCR determination of the BID expression pattern. B, Western blots of the BID protein. C, RT‐qPCR determination of the expression patterns of LINC00472 and miR‐23a‐3p. D, Western blots of BID protein. E, CCK‐8 analysis of cell proliferation. F, Western blots of Ki‐67 and PCNA proteins. G, Flow cytometric analysis of cell apoptosis. Data among multiple groups were compared by one‐way ANOVA with Tukey's post hoc test. *P < 0.05

We silenced LINC00472 and/or overexpressed FOXO3 in the BXPC3 cells to evaluate whether LINC00472 could regulate the BID expression pattern through manipulating FOXO3. RT‐qPCR and Western blot results showed that the expression pattern of BID had declined after silencing LINC00472 but had elevated by overexpressing FOXO3, while overexpressing FOXO3 could obliterate the suppressive effect on BID expression pattern mediated by silencing LINC00472 (Figure 8A,B). Furthermore, we silenced LINC00472 and/or overexpressed BID in the pancreatic cancer cell BXPC3. RT‐qPCR and Western blot results revealed that the BID expression pattern was suppressed, while the expression of miR‐23a‐3p was increased after individually silencing LINC00472. The expression pattern of BID reduced by silencing LINC00472 was restored by oe‐BID (Figure 8C,D). Cell proliferation and apoptosis were assayed by CCK‐8 and flow cytometry, respectively, while the expression patterns of the proliferation markers Ki‐67 and PCNA were examined by Western blotting. Silencing of LINC00472 could evidently facilitate the cell proliferation and suppress cell apoptosis, and correspondingly, the expression patterns of Ki‐67 and PCNA were increased. However, restoration of BID could counteract the regulatory effects on cell proliferation and apoptosis mediated by silencing LINC00472 (Figure 8E‐G). In summary, the preceding results indicated that silencing of LINC00472 inhibited the expression pattern of BID through miR‐23a‐3p/FOXO3 to promote the proliferation and inhibit the apoptosis of pancreatic cancer cells.

## 4 DISCUSSION

Pancreatic cancer, as one of the most fatal malignancies, presents with low diagnostic sensitivity and poor prognosis.^25^ Clinical statistics have revealed that even after conventional curative resection, the 5‐year survival rate in patients with pancreatic cancer is as low as about 25%.^4^ For the aetiology of pancreatic cancer, in addition to the genetic components, various modifiable environmental components, such as tobacco, alcohol intake and chronic pancreatitis, have been identified as vital risk factors for the development of such disease.^26^ In order to improve the overall survival rate of patients, besides the development of highly sensitive screening programmes,^27^ it is vital to identify the molecular mechanisms to promote the development of novel therapeutic strategies. Our study primarily demonstrated the ability of LINC00472 to competitively bind to miR‐23a‐3p to boost the FOXO3 expression and transcriptionally activate the BID expression for suppression of pancreatic cancer progression.

Initially, repressed transcription of LINC00472 by ZEB1 was identified in pancreatic cancer. Existing research has elicited the functionality of LINC00472 as potential biomarkers for the early detection of pancreatic ductal adenocarcinoma.^28^ Strikingly, the GEO2R analysis data set revealed that LINC00472 was poorly expressed in pancreatic cancer, and consistently, our research validated the presence of such phenotype in both pancreatic cancer cell line and clinical samples, speculating a regulatory role of LINC00472 in pancreatic cancer. Moreover, our findings identified close correlations of the LINC00472 expression with the patient's TNM stage, where a more advanced stage yielded a lower expression. In the light of ZEB1 as an EMT marker,^29^ a comprehensive report determined its interaction with lncRNAs to facilitate metastasis and progression of pancreatic cancer,^30^ thus speculating that ZEB1 was apparently involved in the signalling axis initiated by LINC00472. Our findings identified that ZEB1 expression was significantly negatively correlated with the LINC00472 expression pattern in clinical pancreatic cancer tissues, where overexpression of ZEB1 inhibited the LINC00472 expression in the pancreatic cancer cells, and was validated by means of dual‐luciferase reporter assay. Collectively, our data suggested that the LINC00472 expression was suppressed by ZEB1 in the pancreatic cancer tissues and cells. Existing literature has elicited the ability of LINC00472 to suppress the progression of various types of cancer, including gastric cancer^31^ and breast cancer.^32^ Herein, we experimentally demonstrated the antitumour activity of LINC00472 in pancreatic cancer as an evident overexpression of LINC00472 inhibited the proliferation and promoted the apoptosis of pancreatic cancer cells while inhibiting tumorigenesis in vivo.

Furthermore, our study suggested that LINC00472 could competitively bind to miR‐23a‐3p to up‐regulate the FOXO3 expression. Extensive research has elicited fundamental biological functions and clinical applications of miRNAs in multiple types of cancer.^33^ In the light of LINC00472 serving as ceRNA of miRNAs, such as miR‐24 and miR‐93‐5p to regulate the manifestation and development of severe diseases including atrial fibrillation and hepatocellular carcinoma,^34^, ^35^ we speculated that LINC00472 may regulate the progression of pancreatic cancer through integral binding to specific miRNA, where RNA pull‐down assay demonstrated the ability of LINC00472 to bind to miR‐23a‐3p. Subsequently, we predicted FOXO3 as a downstream target of miR‐23a‐3p in pancreatic cancer through the starBase database. Their binding relationship was ascertained by RNA pull‐down, while the binding site was validated by dual‐luciferase reporter gene assay. Moreover, miR‐23a‐3p has been reported to function as an oncogenic regulator of pancreatic cancer.^12^ Existing literature has reported that FOXO3 is poorly expressed in pancreatic cancer, thus signifying its vital significance in tumorigenesis and cancer development,^36^ which was consistent with our finding. Essentially, miR‐23a‐3p could evidently target the downstream target gene so as to regulate cancer progression.^37^ FOXO3 was reported to suppress pancreatic cancer progression,^38^ where its knockdown facilitated the metastasis of pancreatic ductal adenocarcinoma by inducing EMT.^39^ To validate the tumour‐suppressive role of FOXO3 in pancreatic cancer, we conducted gain‐of‐function experiments, the results of which suggested that FOXO3 overexpression could suppress the proliferation of pancreatic cancer cells alongside inducing their apoptosis. Furthermore, our data revealed that LINC00472 silencing facilitated the progression of pancreatic cancer cells by inhibition of FOXO3 in vitro, while such interaction was evident during the tumorigenesis in vivo. Finally, in our study, BID was initially profiled as a downstream factor of FOXO3, and the results of dual‐luciferase reporter gene assay suggested that FOXO3 could target the promoter region of BID and radically induce its expression. As the critical roles of BID were identified in initiating the apoptosis of pancreatic cancer cells,^15^ we suggested that LINC00472 may up‐regulate the expression of BID through FOXO3 to induce the apoptosis of pancreatic cancer. Such regulatory axis was verified in both in vitro and in vivo settings, and our data demonstrated that LINC00472 silencing had fundamentally suppressed BID expression through miR‐23a‐3p‐mediated inhibition of FOXO3 to promote proliferation and inhibit the apoptosis of pancreatic cancer cells.

**FIGURE 9:**
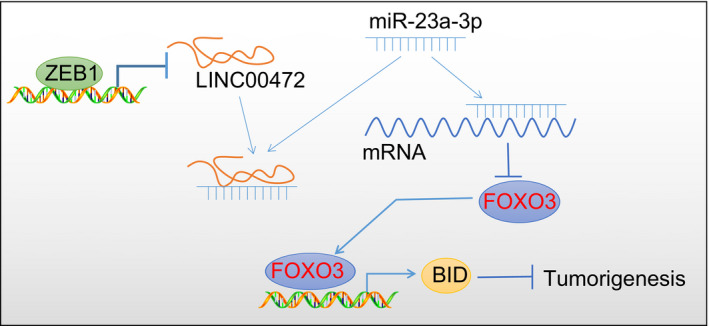
Molecular mechanism mediated by LINC00472 in the progression of pancreatic cancer. LINC00472 promotes the expression pattern of FOXO3 by competitively binding to miR‐23a‐3p, and FOXO3 curbs the progression of pancreatic cancer by binding to the promoter region of the BID gene to enhance its expression pattern. ZEB1, as a transcription factor, can bind to the promoter region of LINC00472 to suppress its expression pattern

To conclude, our data presented that LINC00472, a lncRNA down‐regulated by ZEB1 in pancreatic cancer, could function as tumour suppressor in pancreatic cancer, thus establishing the significance of LINC00472 as a therapeutic target against pancreatic cancer. Essentially, the identification of the regulatory axis that LINC00472 competitively binds to miR‐23a‐3p to induce FOXO3 and activate BID contributes to a comprehensive understanding of the underlying mechanisms involved in tumorigenesis and progression (Figure 9).

## CONFLICT OF INTERESTS

The authors declare that they have no conflicts of interest.

## AUTHOR CONTRIBUTIONS

**Cong Bi:** Conceptualization (equal); Data curation (equal); Formal analysis (equal); Funding acquisition (equal); Investigation (equal); Methodology (equal); Visualization (equal); Writing‐original draft (equal); Writing‐review & editing (equal). **Gang Wang:** Conceptualization (equal); Data curation (equal); Formal analysis (equal); Funding acquisition (equal); Investigation (equal); Methodology (equal); Writing‐original draft (equal); Writing‐review & editing (equal).

## ETHICS APPROVAL AND CONSENT TO PARTICIPATE

All research procedures were conducted with approval of the Ethics Committee of The Fourth Affiliated Hospital of China Medical University and in line with the *Declaration of Helsinki*. All patients and/or legal guardians signed the informed consent documentation prior to experiments. All animal experiments were approved by the Animal Experiment Ethics Committee of The Fourth Affiliated Hospital of China Medical University and conducted in accordance with the Guide for the Care and Use of Laboratory Animals issued by US National Institutes of Health. Great efforts were made to minimize the number of animals used in the experiments and their suffering.

## Supporting information

Table S1

## Data Availability

The data sets generated during the current study are available.
