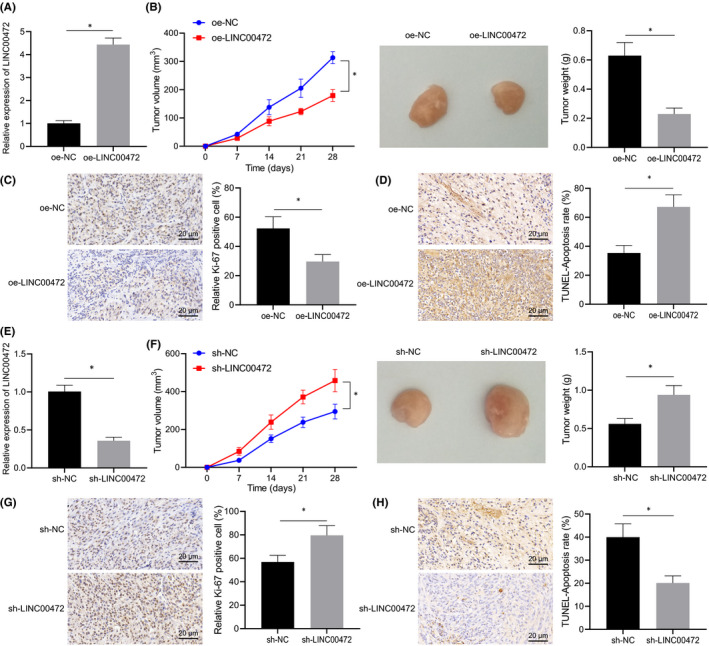# Corrigendum

**DOI:** 10.1111/jcmm.17327

**Published:** 2022-07-06

In Cong Bi et al,^1^ in Figure 3b, the direction of the pictures was reversed. Therefore, during typesetting, the results OE NC and oe‐linc00472 were wrongly typeset. The correct Figure 3 is shown below. The authors confirm that all results and conclusions of this article remain unchanged.