# Supplementary material for: LINC00472 suppressed by ZEB1 regulates the miR‐23a‐3p/FOXO3/BID axis to inhibit the progression of pancreatic cancer
Source: J Cell Mol Med. 2021 Aug 7;25(17):8312–28. doi: 10.1111/jcmm.16784 (PMC8419165; doi:10.1111/jcmm.16784)
Supplement: Supplementary file 1 — Table S1 [file JCMM-25-8312-s001.docx]

**Supplementary Table 1.** Clinicopathological characteristics of patients with pancreatic cancer

| Clinicopathological characteristics | n |
| --- | --- |
| Age (year) | |
| ≥ 60 | 20 |
| < 60 | 50 |
| Gender | |
| Male | 42 |
| Females | 28 |
| Tumor diameter (cm) | |
| ≤ 4 | 45 |
| > 4 | 25 |
| CA19-9 | |
| Positive | 60 |
| Negative | 10 |
| CEA | |
| Positive | 50 |
| Negative | 20 |
| Clinical stage | |
| IA | 3 |
| IB | 20 |
| IIA | 7 |
| IIB | 10 |
| III | 20 |
| IV | 10 |
